# Anticancer property of Hemp Bioactive Peptides in Hep3B liver cancer cells through Akt/GSK3β/β‐catenin signaling pathway

**DOI:** 10.1002/fsn3.1976

**Published:** 2021-02-09

**Authors:** Lian‐Hui Wei, Yan Dong, Yu‐Feng Sun, Xue‐Song Mei, Xue‐Song Ma, Jie Shi, Qing‐li Yang, Yan‐Ru Ji, Zheng‐Hai Zhang, Hu‐Nan Sun, Xing‐Rong Sun, Shu‐Min Song

**Affiliations:** ^1^ Daqing Branch of the Heilongjiang Academy of Sciences Daqing China; ^2^ Daqing Branch of the Heilongjiang Academy of Agricultural Sciences Daqing China; ^3^ College of Life Science and Technology Heilongjiang Bayi Agricultural University Daqing China

**Keywords:** anticancer, GSK3β signaling, hemp seed (*Cannabis sativa L.*), hydrolysates, ROS

## Abstract

Foodborne protein hydrolysates exhibit biological activity that may be therapeutic in a number of human disease settings. Hemp peptides (HP) generated by controlled hydrolysis of hemp proteins have a number of health benefits and are of pharmaceutical value. In the present study, we produce small molecular weight HP from hemp seed and investigate its anticancer properties in Hep3B human liver cancer cells. We demonstrate that HP treatment increased apoptosis, reduced cell viability, and reduced cell migration in Hep3B human liver cancer cells without affecting the normal liver cell line L02. We correlate these phenotypes with increased cellular ROS levels, upregulation of cleaved caspase 3 and Bad, and downregulation of antiapoptotic Bcl‐2. HP treatment led to increased Akt and GSK‐3β phosphorylation, with subsequent downregulation of β‐catenin, suggesting β‐catenin signaling modulation as a critical mechanism by which HP exhibits anticancer properties. Our findings suggest HP are of potential therapeutic interest for liver cancer treatment.

## Graphical abstract

Anti‐cancer property of new hemp protein hydrolysates on human liver cancers

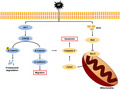

## 1 INTRODUCTION

*Cannabis sativa L*., commonly known as hemp, is an annual herbaceous plant belonging to the Cannabaceae family, which is cultivated in China and Canada (ElSohly et al., 2017; Jian et al., 2018). Hemp has been an important source of raw materials in manufacturing and industry, including use of the hemp seeds, fiber, meal, and oil (Grof, 2018; Potter, 2014). Industrial hemp seeds are a novel variety of cannabis which contain low levels of δ‐9‐tetrahydrocannabinol (THC), with a concentration of less than 0.3%; industrial hemp seeds are now legally grown across dozens of countries worldwide (Mechoulam & Gaoni, 1967) and have been used in the development of numerous products, including clothing, chemicals, bioenergy, and food health, among others (Zengin et al., 2018). The medicinal properties of hemp seeds have been documented for some time and include inhibition LPS‐activated primary human monocytes inflammatory response (for a detailed list see the 2000 edition of the “Pharmacopoeia of the People's Republic of China” and the 2001 edition of “Medicinal and Food Homologous”) (Bonini et al., 2018). The oil extracted from hemp seeds is rich in polyunsaturated fatty acids, proteins, and essential amino acids (Malomo et al., 2014).

Protein hydrolysates, produced by conversion of protein to peptides, have demonstrated a range of biological health effects both in vitro and in vivo, including antioxidant, antihypertensive, antimicrobial, antithrombotic, hypocholesterolemic, mineral‐binding, anticoagulant, immunomodulatory, anti‐inflammatory, and cyto‐modulatory properties. Moreover, several such peptides have been demonstrated to possess more than one of these properties. Over the past decade, exploration and use of hemp peptides (HP) for their health benefitting properties have been proposed. A number of studies have identified specific benefits of HP, including angiotensin‐converting enzyme (ACE) inhibition (Orio et al., 2017), antioxygenation (Atalay et al., 2019; Teh et al., 2016), anticholinesterase (AChE) activity (Asaduzzaman et al., 2014; Yan et al., 2015), cholesterol regulation (Zanoni et al., 2017), and regulation of serum glucose levels (Najafipour & Beik, 2016).

Liver cancer is a common malignant tumor of adulthood and represents a major cause of morbidity and mortality worldwide (Zhou et al., 2016). Liver cancer is rarely diagnosed at early stage; cases typically present with hepatic disease, cirrhosis, and poor hepatic function symptomatic of advanced stage cancer (Lin et al., 2014). First‐line treatment for liver cancer comprises cytotoxic chemotherapy which is associated with serous side effects, including agents such as 5‐fluorouracil (5‐FU), cisplatin, and shikonin (Bruix et al., 2016). Hence, novel effective anticancer therapies with fewer side effects remain an area of intense research interest in liver cancer treatment.

Among their other biological activities, peptides have demonstrated anticancer activity; peptides harbor higher specificity and affinity in comparison with conventional medicines and are associated with fewer side effects while demonstrated greater tissue penetration (Li et al., 2014). Recently, a number of studies have demonstrated that peptide‐like molecules such as dietary proteins and amino acids display anti‐inflammatory, antioxidation, antiproliferative, and anticancer properties; such molecules therefore represent novel candidate drugs for future clinical trials (Anwanwan et al., 2020). Specifically, maize peptides, mung bean peptides, and soybean peptides have been shown to induce cancer cell apoptosis, autophagy, or cell cycle arrest (Dia & Mejia, 2010; Hernández‐Ledesma et al., 2013; McConnell et al., 2015; de Mejia & Dia, 2010; Soucek et al., 2006). However, the anticancer properties of HP in the context of liver cancer are poorly understood.

Here, we investigate the effect of protein hydrolysate HP on liver cancer cells; we measure the impact of HP treatment on liver cancer cell viability, migration, mitochondrial membrane potential, and reactive oxygen species (ROS) levels, making use of the Hep3B human liver cancer cell line. We determine the impact of HP treatment on cellular signaling pathways, including classical GSK‐3β/β‐catenin signaling. Our findings improve upon the current understanding of the anticancer properties of HP as novel candidate anticancer molecules.

## 2 MATERIALS AND METHODS

### 2.1 Preparation of hemp peptides

Defatted hemp seed meal was obtained from Liaoning Qiaopai Biotech Co., Ltd. 20% (w/v, protein basis) aqueous hemp seed protein isolate were soaked in neutral protease (3%w/v, protein basis) and papain (0.4%w/v, protein basis) at pH 5.0 at 70°C for 3 hr, heated to 95°C for 15 min, and centrifuged at 10,000 *g* for 15 min at 4°C. The supernatant was discarded to obtain hemp protein powder (Pap et al., 2020), which was then hydrolyzed to produce hemp protein hydrolysates (Gabotti et al., 2019).

### 2.2 Ultrafiltration

Hemp protein hydrolysates were separated into a low molecular weight fraction by ultrafiltration using the Pellicon^®^2 Mini Cassette (Membrane NMWCO: 1 Ku; EMD Millipore Co.). Soluble peptides were collected using a <1 Ku membrane. Small molecule peptides were freeze‐dried and stored at −20°C prior to use.

### 2.3 Cells culture

Hep3B liver cancer and L02 normal hepatocyte cell lines were cultured in Dulbecco's Modified Eagle's medium (DMEM) (Invitrogen); medium was supplemented with 10% fetal bovine serum (FBS, Hyclone), penicillin (100 U/ml), and streptomycin (100 mg/ml) (P/S) (Solarbio life sciences); cells were incubated at 37°C with 5% CO_2_.

### 2.4 Cell viability assay

Cell viability was assessed by MTT colorimetric assay [3‐(4,5‐dimethylthiazol‐2‐yl)‐2,5 ‐diphenyltetrazolium bromide, Sigma‐Aldrich]. Hep3B and L02 cells were seeded in 96‐well plates at 4 × 10^3^ cells/well and treated with HP (0, 0.5, 1, 2, 5, and 10 mg/ml) for 24 hr. Following treatment, cells were incubated with 10 μl (0.5 mg/ml) of MTT at 37°C in 5% CO_2_ for 2 hr. The supernatant was then removed, and formazan was solubilized with DMSO. Absorbance was measured at 490 nm using the UV MAX kinetic microplate reader platform (Molecular Devices, LLC).

### 2.5 Apoptosis detection with Annexin V‐FITC

Apoptosis levels were determined by Annexin V fluorescence assay. Hep3B and L02 cells were seeded in 6‐well plates at 2 × 10^5^ cells/well and following 0, 6‐, and 12‐hr incubation with 10 mg/ml HP. Levels of apoptosis were measured using the Annexin V‐FITC and PI detection Kit (Solarbio life sciences); signal was detected by fluorescence microscopy (EVOS^®^xl core cell culture microscope, Advanced Microscopy Group, Paisley) and flow cytometry (FACSCalibur, BD Biosciences); data were analyzed using WinMDI version 2.9 (BD Biosciences).

### 2.6 Wound healing assay

Cell migration was assessed by wound healing assay. Hep3B cells were seeded in 6‐well plates at 1 × 10^6^ cells/well. A linear scratch across the cell monolayer was made using small pipette tip. Cells were rinsed three times with PBS prior to addition of HP (0, 2, and 10 mg/ml). Cells were imaged at 0 and 24‐hr timepoints using a fluorescence microscopy (EVOS^®^xl core cell culture microscope, Advanced Microscopy Group, Paisley) to assess migration.

### 2.7 Dectection of ROS production

Cellular and mitochondrial ROS levels of cells were determined using DCFH‐DA (Thermo Fisher Scientific) and MitoSOX (Thermo Fisher Scientific) staining. Hep3B cells were seeded in 6‐well plates at 2 × 10^5^ cells/well and treated with 10 mg/ml HP for 0, 6, and 12 hr. Nuclei were visualized under a microscope following 20‐min incubation with DAPI stain (Solarbio life sciences, Beijing, P.R. China).

### 2.8 Mitochondrial depolarization assays

Mitochondrial membrane potential was assessed by JC‐1 assay (Beyotime). JC‐1 is a membrane‐permeable dye that selectively enters mitochondria; change in mitochondrial membrane potential can be detected by the fluorescence transition of aggregates. Hep3B cells were treated with 10 mg/ml HP for 0, 6, and 12 hr prior to incubation with 20 mM JC‐1 for 15 min at 37°C and then washed with PBS. Nuclei were visualized by DAPI (Solarbio life sciences) staining. After washing with PBS, cells were imaged using a fluorescence core cell culture microscope (EVOS^®^xl core cell culture microscope); fluorescence intensity was assessed qualitatively.

### 2.9 Western blotting analysis

Hep3B cells were seeded in 6‐well plates at 2 × 10^5^ cells/well and treated with 10 mg/ml HP for 0, 1, 3, 6, and 12 hr. Cell protein lysates were separated on 12% SDS‐PAGE (sodium dodecyl sulfate–polyacrylamide gels) and transferred onto nitrocellulose membranes (Millipore). Membranes were blotted with primary antibodies against caspase‐3 (Cell Signaling Technology, #9661), Bad (Cell Signaling Technology, #9268), Bcl‐2 (Cell Signaling Technology, #15071), Akt (Cell Signaling Technology, #2920), p‐Akt (Cell Signaling Technology, #4060), Gsk‐3β (Beyotime, #AG751), p‐Gsk‐3β (Beyotime, #AG753), β‐catenin (Beyotime, #AC106), p‐β‐catenin (Bioss, #bs‐3085), and β‐actin (Cell Signaling Technology, #3700) (dilution 1:2,000) at 4°C overnight. Membranes were washed five times with Tris‐buffered saline containing Tween‐20 (TBST) (10 mM Tris‐HCl (pH 7.5), 150 mM NaCl, and 0.2% Tween‐20) and were subsequently incubated with Horseradish Peroxidase‐conjugated goat antirabbit IgG (Sangon Biotech) or antimouse IgG (Sangon Biotech) for 1 hr at room temperature (RT). After the removal of excess antibodies by washing with TBST, signal detection was performed using chemiluminescence (GE Healthcare Life Sciences) according to the manufacturer's instructions.

### 2.10 Statistical analysis

Data from at least three independent experiments were described as the means ± standard deviation (*SD*). Repeated measures analysis of variance (ANOVA) was used to analyze changes across time and differences between groups in each experiment. Unpaired two‐tailed Student's *t* test was used to determine statistical significance between groups. *p* < .05 was considered statistically significant （*p < .05; **p < .01; ***p < .001).

## 3 RESULT

### 3.1 Hemp peptides preparation and identification

**Figure 1:**
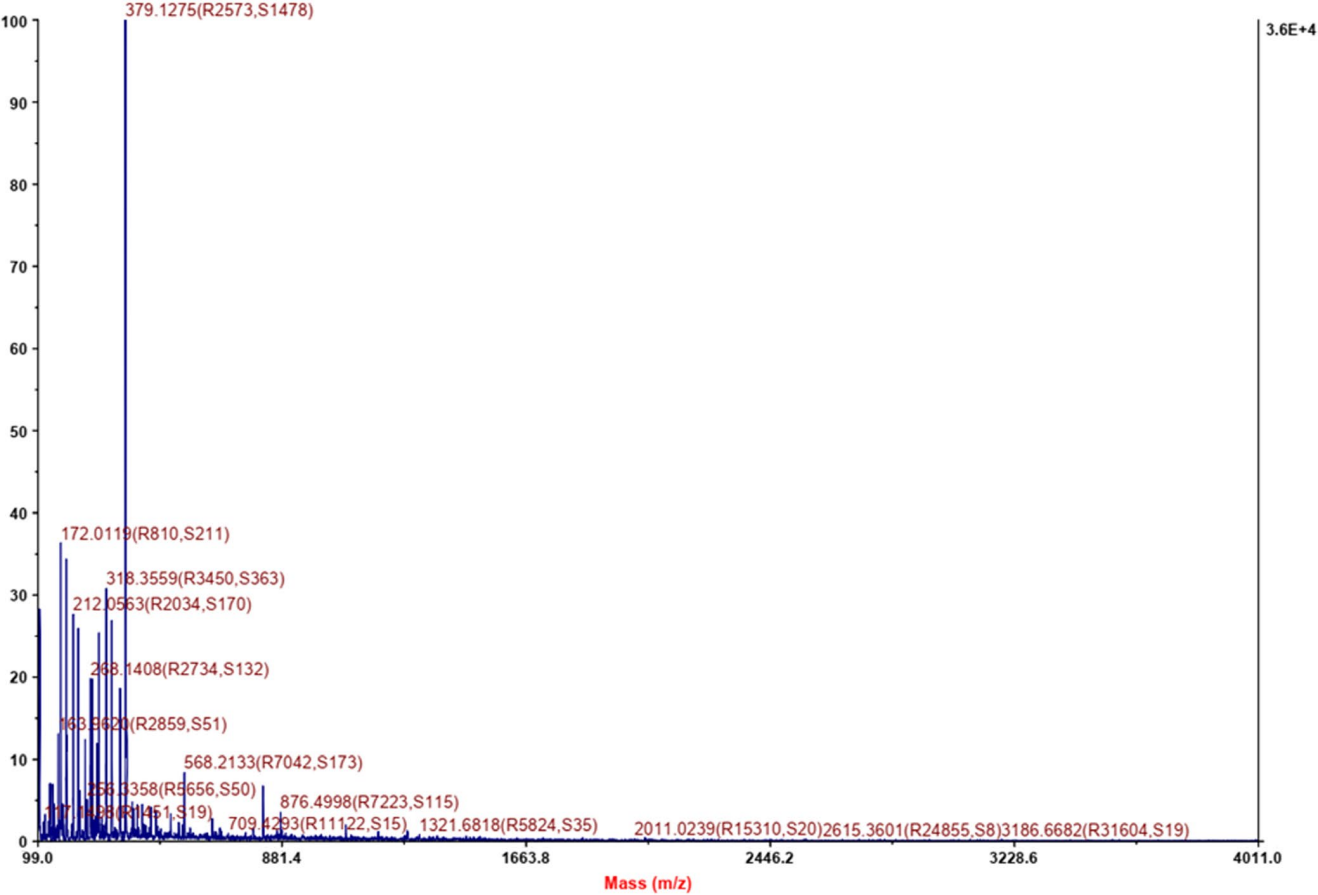
Molecular weight (M.W.) distribution of hemp peptides estimated by MALDI‐TOF/TOF mass spectrometry

Hemp protein underwent hydrolysis by papain and neutral protease, yielding HP. Assessment of molecular weight distribution by MALDI‐TOF/TOF mass spectrometry indicated a distribution range of 170–900 ku. The bulk of peptides were <400 ku (Figure 1), indicating the obtained HP mainly comprise 2–3 amino acid residues. It has been reported that short oligopeptides, especially dipeptides or tripeptides, are easily absorbed and utilized by the human body (McGraw et al., 2014); these low molecular weight HP therefore represent those most likely to be absorbed and exert their biological activity.

### 3.2 Effect of hemp peptides on apoptosis and migration in Hep3B liver cancer cells

**Figure 2:**
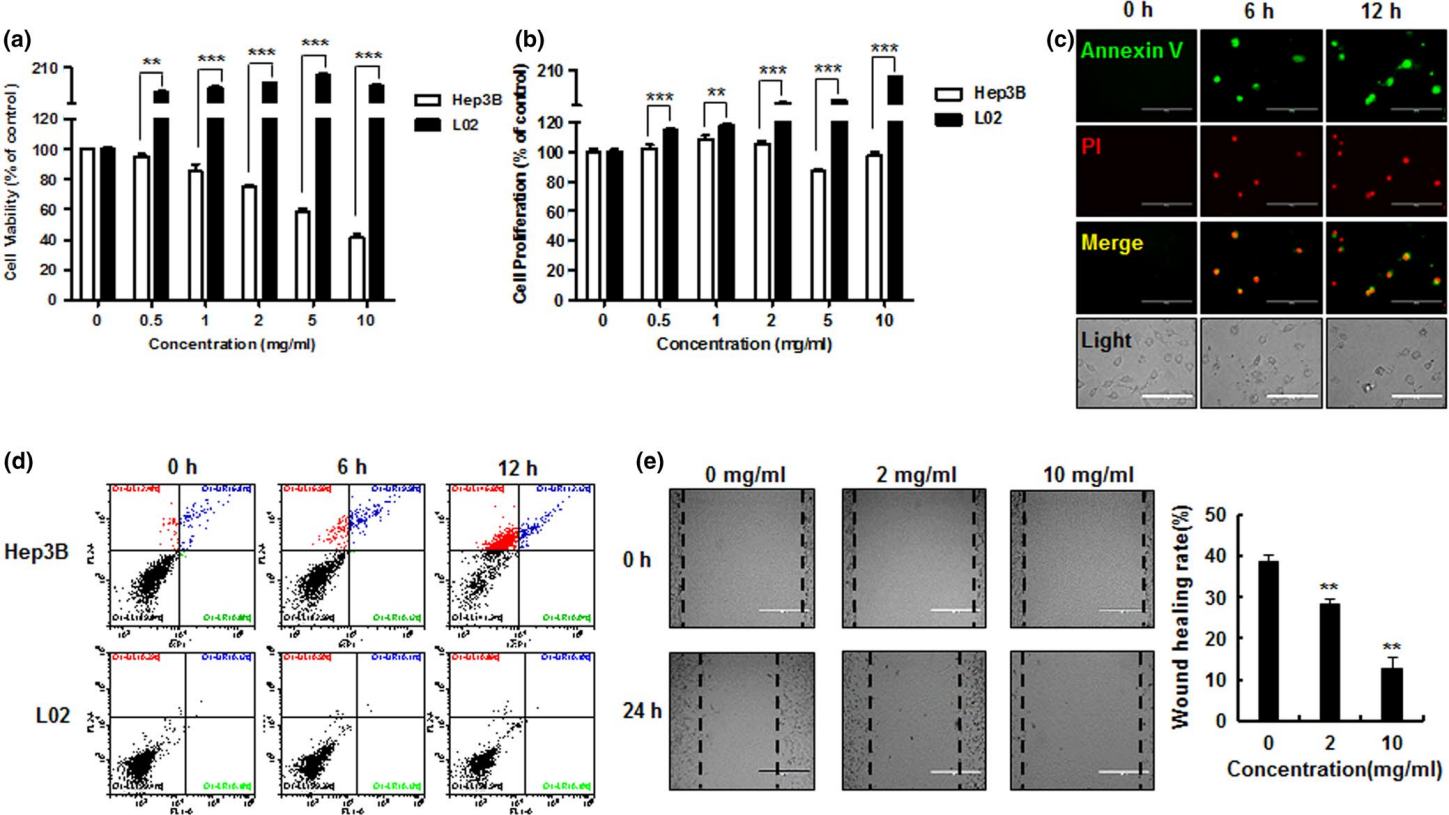
Effect of hemp peptides on cell viability, cell proliferation, apoptosis, and cell migration in Hep3B cells. Hep3B and L02 cells were treated with HP (0, 0.5, 1, 2, 5, and 10 mg/ml) for 24 hr. (a) Cell viability and (b) cell proliferation were measured by MTT colorimetric assay. (c) Hep3B cells were treated with 10 mg/ml HP for 0, 6, and 12 hr. Apoptosis was assessed by Annexin V‐FITC(green)/PI (red) fluorescence microscopy. (d). Hep3B cells and L02 cells were treated with 10 mg/ml HP for 0, 6, and 12 hr. Rate of apoptosis was analyzed by flow cytometry. (e). Hep3B cell migration assessed by wound healing assay after treatment with 0, 2, and 10 mg/ml hemp peptides (left panel); healing rate is shown together after hemp peptide treatment (right panel). Scale bars represent 100 μm; values are given as the mean of at least three independent experiments ± *SEM* (**p < .01, ***p < .001)

Hep3B liver cancer cells and comparator L02 normal liver cells were treated with varying HP concentrations for 24 hr. The cellular viability and proliferation (using 1% and 10% FBS, respectively) were measured by MTT assay. HP treatment effectively reduced both cellular viability and proliferation in Hep3B cancer cells, while the L02 normal liver cell line was not affected (Figure 2a,b): 10 mg/ml HP induced cell death in >50% of Hep3B cells after 24 hr of exposure. HP treatment lead to a significant time‐dependent increase in apoptosis in Hep3B cells (Figure 2c,d), with no effect seen in the L02 comparator cells, suggesting selective cytotoxicity. HP treatment also significantly inhibited Hep3B cell migration in a concentration‐dependent manner as determined by wound healing assay (Figure 2e).

### 3.3 Cellular ROS levels plays major roles in HP‐induced liver cancer cell apoptosis

**Figure 3:**
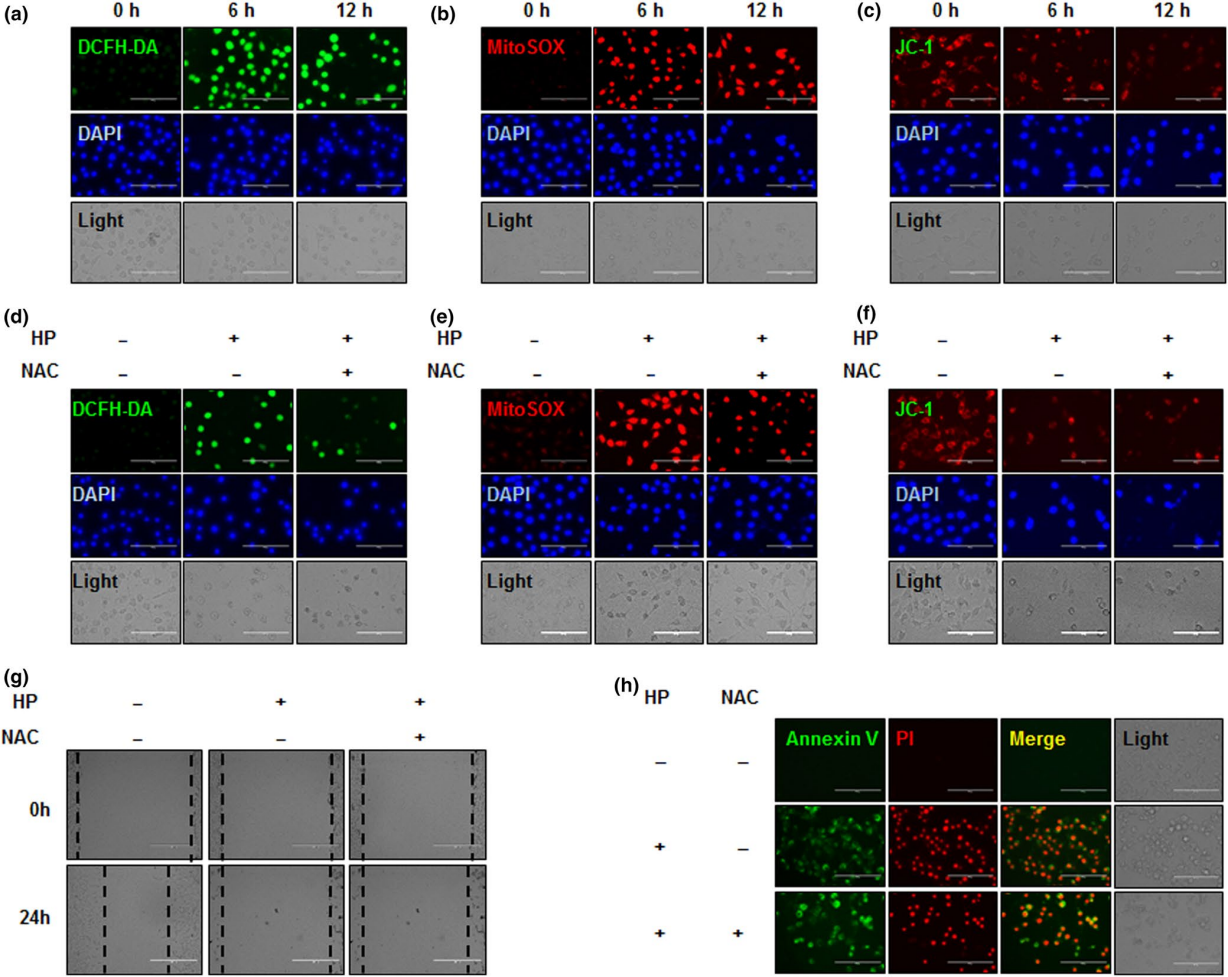
Effect of hemp peptides on cellular and mitochondrial reactive oxygen species and mitochondrial membrane permeability in Hep3B cells. Hep3B cells were treated with 10 mg/ml hemp peptides for 0, 6, and 12 hr. (a) Cellular reactive oxygen species (ROS) levels were detected by DCFH‐DA (green) and DAPI (blue) staining. (b) Mitochondrial ROS levels were measured by fluorescence microscopy using MitoSOX (red) and DAPI (blue) staining. (c) Mitochondrial membrane potential (MMP) was detected by JC‐1 assay. Hep3B cells were pretreatment with 5 mM NAC for 30 min prior to 10 mg/ml hemp peptide treatment for 12 hr. (d) Cellular ROS levels determined by DCFH‐DA (green) and DAPI (blue) staining. (e) ROS levels at the mitochondria were measured by fluorescence microscopy using MitoSOX (red) and DAPI (blue) staining. (f) Mitochondrial membrane potential detected by JC‐1 assay. (g) Cell migration determined by wound healing assay. (H). Apoptosis levels determined by fluorescence microscopy of Annexin V‐FITC (green) and PI (red). Scale bars represent 100 μm for all images

Changes to intracellular and mitochondrial ROS levels were determined by DCFH‐DA/DAPI and MitoSOX/DAPI fluorescence imaging. Treatment of Hep3B cells with HP resulted in increased intracellular and mitochondrial ROS levels over time (Figure 3a,b). HP also significantly increased MMP as determined by JC‐1 assay (Figure 3c). Pretreatment of Hep3B cells with 5mM N‐Acetylcysteine (NAC) for 30 min decreased HP‐induced ROS and MMP elevation (Figure 3d–f) and decreased HP‐induced effects on migration and apoptosis (Figure 3g,h).

### 3.4 HP induces apoptosis through mitochondria‐dependent and Akt/GSK3β/β‐catenin signaling pathways in Hep3B cells

**Figure 4:**
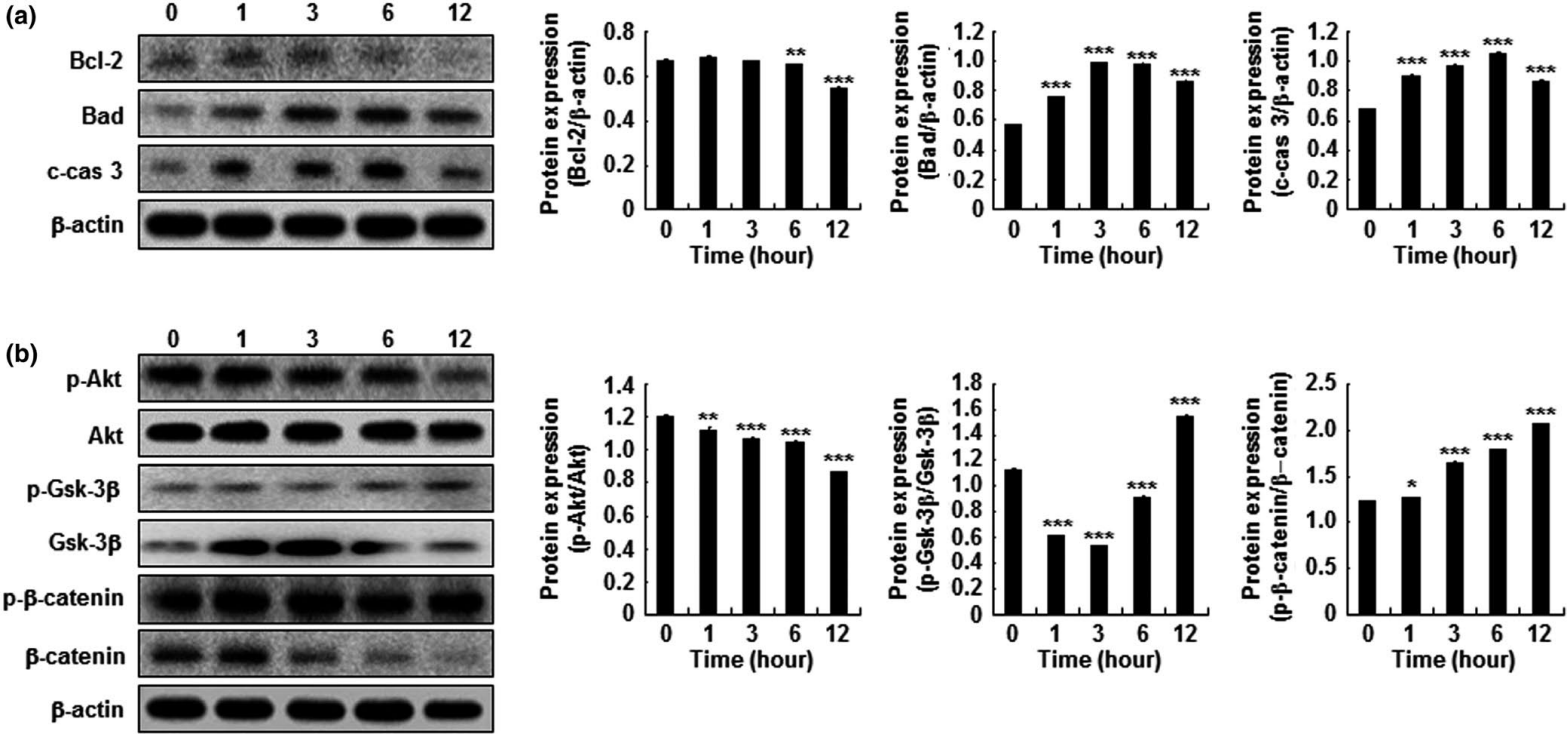
Effect of hemp peptides on apoptosis, apoptosis‐related proteins expression, and Akt/GSK3β/β‐catenin signaling in Hep3B cells. Western blots of cell lysates following treatment with 10 mg/ml hemp peptides. (a) Proapoptotic proteins Bad and cleaved caspase 3 (c‐cas 3) alongside antiapoptotic Bcl‐2. (b) p‐AKT, AKT, p‐Gsk‐3β Gsk‐3β, p‐β‐catenin, and β‐catenin protein levels in Hep3B cells. β‐actin was used as a loading control. Data are presented as the mean of three samples ± *SEM* (*p < .05, **p < .01, ***p < .001)

Caspases and Bcl‐2 protein family members were investigated by Western blot as key players in mitochondrial‐dependent cell death pathways. HP‐treated Hep3B cells demonstrated upregulation of proapoptotic Bad and cleaved caspase 3, while the antiapoptotic protein Bcl‐2 was downregulated (Figure 4a). HP treatment also induced a significant reduction in Akt phosphorylation, while GSK‐3β and β‐catenin phosphorylation was increased (Figure 4b).

## 4 DISCUSSION

Protein hydrolysates have become the focus of substantial research interest due to their therapeutic properties in multiple disease settings, including inflammatory and metabolic diseases. Such hydrolysates have also demonstrated anticancer properties. 1 ku molecular weight donkey serum albumin peptides, produced by hydrolysis, have been demonstrated to inhibited cell proliferation in human leukemia, liver cancer and breast cancer cells, suggesting these molecules may be of therapeutic interest in the context of cancer treatment. It has also been reported that cannabis sativa L.ethanolic extract can be useful as a treatment for skin inflammation (McGraw et al., 2014) and that cannabinoids can induce apoptosis in pancreatic cancer (Carracedo et al., 2006) and breast cancer (Shrivastava et al., 2011) cells through endoplasmic stress and ROS elevation. Hemp extract has been shown to inhibit the proliferation and growth of prostate cancer (De Petrocellis et al., 2013) and melanoma (Blázquez et al., 2006)cells, and hemp derivatives are able to alter mitochondrial permeability of rat liver cells and increase cytochrome c release (Zaccagnino et al., 2012), suggesting that hemp peptides represent candidate anticancer agents. It has also been reported that the biological activities of peptides are modulated by molecular weight, with low molecular weight correlating with increased activity (Dugnol & Riera, 2016; Li et al., 2019).

In this study, we investigate the potential anticancer properties of small molecular weight HP produced from hemp seed meal. Anticancer agents frequently act via induced of apoptosis in cancer cells, and this has been demonstrated as an important mechanism by which other peptide types exert anticancer activity; rapeseed protein source peptide has been shown to induce apoptosis in HUVEC cells via downregulation of Bcl‐2 and increased cleaved caspase‐3 and Bax expression (Xu et al., 2018). We demonstrate that HP treatment induces apoptosis in Hep3B liver cancer cells, while L02 cells—a cell line representing normal liver cells—are unaffected. We identify increased Bad and cleaved caspase‐3 following HP treatment, with a corresponding decrease in antiapoptotic Bcl‐2 expression. These data provide preliminary biological mechanisms by which HP induced apoptosis in Hep3B cells.

Cellular ROS levels are an important determinant of cell proliferation, survival, angiogenesis, and metastasis in cancer cells (Khan et al., 2015,2016). Abnormal accumulating of ROS leads to the changes in MMP, endoplasmic reticulum stress, membrane lipid peroxidation, protein denaturation, and DNA damage, resulting in decreased cell viability (Liu et al., 2020). Similarly, ROS levels can modulate cancer cell migration, including via intracellular ROS changes in response to cancer treatment (Malfettone et al., 2017). We demonstrate that HP treatment significantly increases cellular ROS levels and correlate this with reduce migration and colony formation in Hep3B cells. Moreover, scavenging of ROS with NAC pretreatment rescues the phenotypes we observe in response to HP, strongly suggesting increased ROS levels act as a major mechanism by which HP exerts anticancer activity.

The Wnt/β‐catenin signaling pathway regulates a large number of cellular activities, including proliferation, differentiation, migration, and cell apoptosis (Nusse & Clevers, 2017). Aberrant Wnt/β‐catenin activation has been identified as a driver event in a number of cancer types; accumulation of cytoplasmic β‐catenin and subsequent entry into the nucleus leads to activation of the TCF/LEF transcriptional program, promoting cell survival (Daneman et al., 2009). Previous studies have shown that knockdown of SOX9 in human lung carcinoma cells induces apoptosis via decreased p‐Wnt and β‐catenin expression (Guo et al., 2018)and that β‐catenin is a pivotal target for glioma and ovarian cancer therapy (Arend et al., 2013; He et al., 2019). It has also been reported that prodigiosin inhibits the phosphorylation of GSK‐3β, restraining β‐catenin‐mediated Wnt activation and subsequent transcriptional activation of prosurvival genes, such as cyclin D1, slowing tumor progression in a breast cancer tumor model mice (Wang et al., 2016). Phosphorylated Akt has the ability to phosphorylate GSK‐3β as a key molecule in this process, which in turn inhibits cell survival via β‐catenin phosphorylation and induction of apoptosis (Liu et al., 2019). The β‐catenin pathway is abnormally activated in liver cancer and has been implicated in tumor formation, progression, metastasis, and drug resistance (Vilchez et al., 2016). A large number of anticancer agents targeted at β‐catenin have been developed, but few have entered clinical trials (Nambotin et al., 2011; Qin et al., 2013; Wei et al., 2011). We demonstrate that HP treatment leads to inactivation of the Akt/GSK3β/β‐catenin signaling pathway via AKT phosphorylation, GSK‐3β inactivation, and subsequent phosphorylation and degradation of β‐catenin.

Together, these data demonstrate that HP from hemp seed protein hydrolysates display anticancer properties. Increase in cellular ROS levels represents a crucial mechanism by which HP exerts its antiproliferative and proapoptotic activity and HP treatment modulates activity of the Akt/GSK/β‐catenin signaling pathway. Our findings suggest that HP represents a promising anticancer therapy in the context of liver cancer; further investigation of the anticancer properties of foodborne protein hydrolysates is warranted.

## CONFLICT OF INTEREST

The authors declare no conflicts of interest.

## AUTHOR CONTRIBUTIONS

Lian‐Hui Wei, Yan Dong, Yu‐Feng Sun, Xue‐Song Mei, and Xue‐Song Ma contributed to data curation; Jie Shi, Qing‐li Yang, Hu‐Nan Sun, Yan‐Ru Ji, and Zheng‐Hai Zhang contributed to formal analysis; Shu‐Min Song contributed to funding acquisition; Lian‐HuiWei and Shu‐Min Song contributed to supervision.

## Data Availability

Research data are not shared.

## References

[fsn31976-bib-0001] Anwanwan, D. , Singh, S. K. , Singh, S. , Saikam, V. , & Singh, R. (2020). Challenges in liver cancer and possible treatment approaches. Biochimica et Biophysica Acta ‐ Reviews on Cancer, 1873(1), 188314. 10.1016/j.bbcan.2019.188314 31682895 PMC6981221

[fsn31976-bib-0002] Arend, R. C. , Londoño‐Joshi, A. I. , Straughn, J. M. Jr , & Buchsbaum, D. J. (2013). The Wnt/β‐catenin pathway in ovarian cancer: A review. Gynecologic Oncology, 131(3), 772–779. 10.1016/j.ygyno.2013.09.034 24125749

[fsn31976-bib-0003] Asaduzzaman, M. , Uddin, M. J. , Kader, M. A. , Alam, A. H. , Rahman, A. A. , Rashid, M. , Kato, K. , Tanaka, T. , Takeda, M. & Sadik, G. (2014). In vitro acetylcholinesterase inhibitory activity and the antioxidant properties of Aegle marmelos leaf extract: Implications for the treatment of Alzheimer's disease. Psychogeriatrics, 14(1), 1–10. 10.1111/psyg.12031 24646308

[fsn31976-bib-0004] Atalay, S. , Jarocka‐Karpowicz, I. , & Skrzydlewska, E. (2019). Antioxidative and anti‐inflammatory properties of cannabidiol. Antioxidants (Basel), 9(1), 21. 10.3390/antiox9010021 PMC7023045 31881765

[fsn31976-bib-0005] Blázquez, C. , Carracedo, A. , Barrado, L. , José Real, P. , Luis Fernández‐Luna, J. , Velasco, G. , Malumbres, M. , Guzmán, M. , Blázquez, C. , Carracedo, A. , Barrado, L. , José Real, P. , Luis Fernández‐Luna, J. , Velasco, G. , Malumbres, M. , & Guzmán, M. (2006). Cannabinoid receptors as novel targets for the treatment of melanoma. The FASEB Journal, 20(14), 2633–2635. 10.1096/fj.06-6638fje 17065222

[fsn31976-bib-0006] Bonini, S. A. , Premoli, M. , Tambaro, S. , Kumar, A. , Maccarinelli, G. , Memo, M. , & Mastinu, A. (2018). Cannabis sativa: A comprehensive ethnopharmacological review of a medicinal plant with a long history. Journal of Ethnopharmacology, 227, 300–315. 10.1016/j.jep.2018.09.004 30205181

[fsn31976-bib-0007] Bruix, J. , Reig, M. , & Sherman, M. (2016). Evidence‐based diagnosis, staging, and treatment of patients with hepatocellular carcinoma. Gastroenterology, 150(4), 835–853. 10.1053/j.gastro.2015.12.041 26795574

[fsn31976-bib-0008] Carracedo, A. , Gironella, M. , Lorente, M. , Garcia, S. , Guzmán, M. , Velasco, G. , & Iovanna, J. L. (2006). Cannabinoids induce apoptosis of pancreatic tumor cells via endoplasmic reticulum stress‐related genes. Cancer Research, 66(13), 6748–6755. 10.1158/0008-5472.Can-06-0169 16818650

[fsn31976-bib-0009] Daneman, R. , Agalliu, D. , Zhou, L. , Kuhnert, F. , Kuo, C. J. , & Barres, B. A. (2009). Wnt/beta‐catenin signaling is required for CNS, but not non‐CNS, angiogenesis. Proceedings of the National Academy of Sciences of the United States of America, 106(2), 641–646. 10.1073/pnas.0805165106 19129494 PMC2626756

[fsn31976-bib-0010] de Mejia, E. G. , & Dia, V. P. (2010). The role of nutraceutical proteins and peptides in apoptosis, angiogenesis, and metastasis of cancer cells. Cancer and Metastasis Reviews, 29(3), 511–528. 10.1007/s10555-010-9241-4 20714786

[fsn31976-bib-0011] De Petrocellis, L. , Ligresti, A. , Schiano Moriello, A. , Iappelli, M. , Verde, R. , Stott, C. G. , & Di Marzo, V. (2013). Non‐THC cannabinoids inhibit prostate carcinoma growth in vitro and in vivo: Pro‐apoptotic effects and underlying mechanisms. British Journal of Pharmacology, 168(1), 79–102. 10.1111/j.1476-5381.2012.02027.x 22594963 PMC3570006

[fsn31976-bib-0012] Dia, V. P. , & Mejia, E. G. (2010). Lunasin promotes apoptosis in human colon cancer cells by mitochondrial pathway activation and induction of nuclear clusterin expression. Cancer Letters, 295(1), 44–53. 10.1016/j.canlet.2010.02.010 20206442

[fsn31976-bib-0013] Dugnol, J. , & Riera, F. A. (2016). Hyperimmunised bovine milk and whey: Influence of pH and enzymatic treatments on the antigen‐binding capacity of immunoglobulin G. Journal of the Science of Food and Agriculture, 96(5), 1814–1820. 10.1002/jsfa.7291 26041450

[fsn31976-bib-0014] ElSohly, M. A. , Radwan, M. M. , Gul, W. , Chandra, S. , & Galal, A. (2017). Phytochemistry of cannabis sativa L. Progress in the Chemistry of Organic Natural Products, 103, 1–36. 10.1007/978-3-319-45541-9_1 28120229

[fsn31976-bib-0015] Gabotti, D. , Locatelli, F. , Cusano, E. , Baldoni, E. , Genga, A. , Pucci, L. , Consonni, R. , & Mattana, M. (2019). Cell suspensions of cannabis sativa (var. Futura): Effect of elicitation on metabolite content and antioxidant activity. Molecules, 24(22), 4056. 10.3390/molecules24224056 PMC6891269 31717508

[fsn31976-bib-0016] Grof, C. P. L. (2018). Cannabis, from plant to pill. British Journal of Clinical Pharmacology, 84(11), 2463–2467. 10.1111/bcp.13618 29701252 PMC6177712

[fsn31976-bib-0017] Guo, Y. Z. , Xie, X. L. , Fu, J. , & Xing, G. L. (2018). SOX9 regulated proliferation and apoptosis of human lung carcinoma cells by the Wnt/β‐catenin signaling pathway. European Review for Medical and Pharmacological Sciences, 22(15), 4898–4907. 10.26355/eurrev_201808_15626 30070325

[fsn31976-bib-0018] He, L. , Zhou, H. , Zeng, Z. , Yao, H. , Jiang, W. , & Qu, H. (2019). Wnt/β‐catenin signaling cascade: A promising target for glioma therapy. Journal of Cellular Physiology, 234(3), 2217–2228. 10.1002/jcp.27186 30277583

[fsn31976-bib-0019] Hernández‐Ledesma, B. , Hsieh, C. C. , & de Lumen, B. O. (2013). Chemopreventive properties of Peptide Lunasin: A review. Protein and Peptide Letters, 20(4), 424–432. 23016582

[fsn31976-bib-0020] Jian, F. , Divagar, D. , Mhaiki, J. , Jayas, D. S. , Fields, P. G. , & White, N. D. G. (2018). Static and dynamic methods to determine adsorption isotherms of hemp seed (Cannabis sativa L.) with different percentages of dockage. Food Science & Nutrition, 6(6), 1629–1640. 10.1002/fsn3.744 30258606 PMC6145264

[fsn31976-bib-0021] Khan, M. , Maryam, A. , Qazi, J. I. , & Ma, T. (2015). Targeting apoptosis and multiple signaling pathways with icariside II in cancer cells. International Journal of Biological Sciences, 11(9), 1100–1112. 10.7150/ijbs.11595 26221076 PMC4515820

[fsn31976-bib-0022] Khan, M. , Maryam, A. , Zhang, H. , Mehmood, T. , & Ma, T. (2016). Killing cancer with platycodin D through multiple mechanisms. Journal of Cellular and Molecular Medicine, 20(3), 389–402. 10.1111/jcmm.12749 26648178 PMC4759477

[fsn31976-bib-0023] Li, C. C. , Lee, Y. C. , Lo, H. Y. , Huang, Y. W. , Hsiang, C. Y. , & Ho, T. Y. (2019). Antihypertensive effects of corn silk extract and its novel bioactive constituent in spontaneously hypertensive rats: The involvement of angiotensin‐converting enzyme inhibition. Molecules, 24(10), 1886. 10.3390/molecules24101886 PMC6572293 31100914

[fsn31976-bib-0024] Li, Y. L. , Dai, X. R. , Yue, X. , Gao, X. Q. , & Zhang, X. S. (2014). Identification of small secreted peptides (SSPs) in maize and expression analysis of partial SSP genes in reproductive tissues. Planta, 240(4), 713–728. 10.1007/s00425-014-2123-1 25048445

[fsn31976-bib-0025] Lin, C. L. , Chien, R. N. , Yeh, C. , Hsu, C. W. , Chang, M. L. , Chen, Y. C. , & Yeh, C. T. (2014). Significant renoprotective effect of telbivudine during preemptive antiviral therapy in advanced liver cancer patients receiving cisplatin‐based chemotherapy: A case‐control study. Scandinavian Journal of Gastroenterology, 49(12), 1456–1464. 10.3109/00365521.2014.962604 25283499

[fsn31976-bib-0026] Liu, H. , Liao, W. , Fan, L. , Zheng, Z. , Liu, D. , Zhang, Q.‐W. , Yang, A. , & Liu, F. (2020). Ethanol extract of Ophiorrhiza pumila suppresses liver cancer cell proliferation and migration. Chinese Medicine, 15, 11. 10.1186/s13020-020-0291-4 32021647 PMC6995237

[fsn31976-bib-0027] Liu, Y. , Huang, Y. , Ding, J. , Liu, N. , Peng, S. , Wang, J. , Wang, F. , & Zhang, Y. (2019). Targeting Akt by SC66 triggers GSK‐3β mediated apoptosis in colon cancer therapy. Cancer Cell International, 19, 124. 10.1186/s12935-019-0837-7 31168297 PMC6509835

[fsn31976-bib-0028] Malfettone, A. , Soukupova, J. , Bertran, E. , Crosas‐Molist, E. , Lastra, R. , Fernando, J. , Koudelkova, P. , Rani, B. , Fabra, Á. , Serrano, T. , Ramos, E. , Mikulits, W. , Giannelli, G. , & Fabregat, I. (2017). Transforming growth factor‐β‐induced plasticity causes a migratory stemness phenotype in hepatocellular carcinoma. Cancer Letters, 392, 39–50. 10.1016/j.canlet.2017.01.037 28161507

[fsn31976-bib-0029] Malomo, S. A. , He, R. , & Aluko, R. E. (2014). Structural and functional properties of hemp seed protein products. Journal of Food Science, 79(8), C1512–C1521. 10.1111/1750-3841.12537 25048774

[fsn31976-bib-0030] McConnell, E. J. , Devapatla, B. , Yaddanapudi, K. , & Davis, K. R. (2015). The soybean‐derived peptide lunasin inhibits non‐small cell lung cancer cell proliferation by suppressing phosphorylation of the retinoblastoma protein. Oncotarget, 6(7), 4649–4662. 10.18632/oncotarget.3080 25609198 PMC4467105

[fsn31976-bib-0031] McGraw, N. J. , Napawan, N. , Toland, M. R. , Schulze, J. , Tulk, B. M. , & Krul, E. S. (2014). Partially hydrolyzed soy protein shows enhanced transport of amino acids compared to nonhydrolyzed protein across an intestinal epithelial cell monolayer. Journal of Food Science, 79(9), H1832–1840. 10.1111/1750-3841.12553 25040304

[fsn31976-bib-0032] Mechoulam, R. , & Gaoni, Y. (1967). The absolute configuration of delta‐1‐tetrahydrocannabinol, the major active constituent of hashish. Tetrahedron Letters, 12, 1109–1111. 10.1016/s0040-4039(00)90646-4 6039537

[fsn31976-bib-0033] Najafipour, H. , & Beik, A. (2016). The impact of opium consumption on blood glucose, serum lipids and blood pressure, and related mechanisms. Frontiers in Physiology, 7, 436. 10.3389/fphys.2016.00436 27790151 PMC5061814

[fsn31976-bib-0034] Nambotin, S. B. , Lefrancois, L. , Sainsily, X. , Berthillon, P. , Kim, M. , Wands, J. R. , Chevallier, M. , Jalinot, P. , Scoazec, J.‐Y. , Trepo, C. , Zoulim, F. , & Merle, P. (2011). Pharmacological inhibition of Frizzled‐7 displays anti‐tumor properties in hepatocellular carcinoma. Journal of Hepatology, 54(2), 288–299. 10.1016/j.jhep.2010.06.033 21055837

[fsn31976-bib-0035] Nusse, R. , & Clevers, H. (2017). Wnt/β‐catenin signaling, disease, and emerging therapeutic modalities. Cell, 169(6), 985–999. 10.1016/j.cell.2017.05.016 28575679

[fsn31976-bib-0036] Orio, L. P. , Boschin, G. , Recca, T. , Morelli, C. F. , Ragona, L. , Francescato, P. , Arnoldi, A. & Speranza, G. (2017). New ACE‐inhibitory peptides from hemp seed (Cannabis sativa L.) proteins. Journal of Agriculture and Food Chemistry, 65(48), 10482–10488. 10.1021/acs.jafc.7b04522 29112398

[fsn31976-bib-0037] Pap, N. , Hamberg, L. , Pihlava, J. M. , Hellström, J. , Mattila, P. , Eurola, M. , & Pihlanto, A. (2020). Impact of enzymatic hydrolysis on the nutrients, phytochemicals and sensory properties of oil hemp seed cake (Cannabis sativa L. FINOLA variety). Food Chemistry, 320, 126530. 10.1016/j.foodchem.2020.126530 32222655

[fsn31976-bib-0038] Potter, D. J. (2014). A review of the cultivation and processing of cannabis (Cannabis sativa L.) for production of prescription medicines in the UK. Drug Testing and Analysis, 6(1–2), 31–38. 10.1002/dta.1531 24115748

[fsn31976-bib-0039] Qin, Y. , Lu, Y. , Wang, R. , Li, W. , & Qu, X. (2013). SL1122‐37, a novel derivative of sorafenib, has greater effects than sorafenib on the inhibition of human hepatocellular carcinoma (HCC) growth and prevention of angiogenesis. BioScience Trends, 7(5), 237–244. 10.5582/bst.2013.v7.5.237 24241174

[fsn31976-bib-0040] Shrivastava, A. , Kuzontkoski, P. M. , Groopman, J. E. , & Prasad, A. (2011). Cannabidiol induces programmed cell death in breast cancer cells by coordinating the cross‐talk between apoptosis and autophagy. Molecular Cancer Therapeutics, 10(7), 1161–1172. 10.1158/1535-7163.Mct-10-1100 21566064

[fsn31976-bib-0041] Soucek, J. , Skvor, J. , Poucková, P. , Matousek, J. , Slavík, T. , & Matousek, J. (2006). Mung bean sprout (Phaseolus aureus) nuclease and its biological and antitumor effects. Neoplasma, 53(5), 402–409. 17013534

[fsn31976-bib-0042] Teh, S. S. , Bekhit, A. E. A. , Carne, A. , & Birch, J. (2016). Antioxidant and ACE‐inhibitory activities of hemp (Cannabis sativa L.) protein hydrolysates produced by the proteases AFP, HT, Pro‐G, actinidin and zingibain. Food Chemistry, 203, 199–206. 10.1016/j.foodchem.2016.02.057 26948606

[fsn31976-bib-0043] Vilchez, V. , Turcios, L. , Marti, F. , & Gedaly, R. (2016). Targeting Wnt/β‐catenin pathway in hepatocellular carcinoma treatment. World Journal of Gastroenterology, 22(2), 823–832. 10.3748/wjg.v22.i2.823 26811628 PMC4716080

[fsn31976-bib-0044] Wang, Z. , Li, B. O. , Zhou, L. , Yu, S. , Su, Z. , Song, J. , Sun, Q. I. , Sha, O. U. , Wang, X. , Jiang, W. , Willert, K. , Wei, L. , Carson, D. A. , & Lu, D. (2016). Prodigiosin inhibits Wnt/β‐catenin signaling and exerts anticancer activity in breast cancer cells. Proceedings of the National Academy of Sciences United States of America, 113(46), 13150–13155. 10.1073/pnas.1616336113 PMC5135380 27799526

[fsn31976-bib-0045] Wei, W. , Chua, M. S. , Grepper, S. , & So, S. K. (2011). Soluble Frizzled‐7 receptor inhibits Wnt signaling and sensitizes hepatocellular carcinoma cells towards doxorubicin. Molecular Cancer, 10, 16. 10.1186/1476-4598-10-16 21314951 PMC3050858

[fsn31976-bib-0046] Xu, F. , Zhang, J. , Wang, Z. , Yao, Y. , Atungulu, G. G. , Ju, X. , & Wang, L. (2018). Absorption and metabolism of peptide WDHHAPQLR derived from rapeseed protein and inhibition of HUVEC apoptosis under oxidative stress. Journal of Agriculture and Food Chemistry, 66(20), 5178–5189. 10.1021/acs.jafc.8b01620 29732892

[fsn31976-bib-0047] Yan, X. , Tang, J. , dos Santos Passos, C. , Nurisso, A. , Simões‐Pires, C. A. , Ji, M. , Lou, H. & Fan, P. (2015). Characterization of lignanamides from hemp (Cannabis sativa L.) seed and their antioxidant and acetylcholinesterase inhibitory activities. Journal of Agriculture and Food Chemistry, 63(49), 10611–10619. 10.1021/acs.jafc.5b05282 26585089

[fsn31976-bib-0048] Zaccagnino, P. , D'Oria, S. , Romano, L. L. , Di Venere, A. , Sardanelli, A. M. , & Lorusso, M. (2012). The endocannabinoid 2‐arachidonoylglicerol decreases calcium induced cytochrome c release from liver mitochondria. Journal of Bioenergetics and Biomembranes, 44(2), 273–280. 10.1007/s10863-012-9431-6 22437740

[fsn31976-bib-0049] Zanoni, C. , Aiello, G. , Arnoldi, A. , & Lammi, C. (2017). Hempseed peptides exert hypocholesterolemic effects with a statin‐like mechanism. Journal of Agriculture and Food Chemistry, 65(40), 8829–8838. 10.1021/acs.jafc.7b02742 28931275

[fsn31976-bib-0050] Zengin, G. , Menghini, L. , Di Sotto, A. , Mancinelli, R. , Sisto, F. , Carradori, S. , Cesa, S. , Fraschetti, C. , Filippi, A. , Angiolella, L. , Locatelli, M. , Mannina, L. , Ingallina, C. , Puca, V. , D’Antonio, M. , & Grande, R. (2018). Chromatographic analyses, in vitro biological activities, and cytotoxicity of cannabis sativa l. essential oil: A multidisciplinary study. Molecules, 23(12), 3266 10.3390/molecules23123266 PMC6320915 30544765

[fsn31976-bib-0051] Zhou, Y. , Li, Y. , Zhou, T. , Zheng, J. , Li, S. , & Li, H. B. (2016). Dietary natural products for prevention and treatment of liver cancer. Nutrients, 8(3), 156. 10.3390/nu8030156 26978396 PMC4808884

